# Gait Rehabilitation Using Hybrid Assistive Limb in Patients With Lower Limb Amputation: Protocol for a Single-Arm Clinical Trial

**DOI:** 10.2196/76509

**Published:** 2025-11-11

**Authors:** Makoto Asaeda, Naoya Orita, Masashi Shimada, Yoshifumi Kono, Kiyo Ueda, Takeya Araki, Naoto Fujita, Yukio Mikami

**Affiliations:** 1 Department of Rehabilitation Medicine Hiroshima University Hospital Hiroshima Japan; 2 Division of Rehabilitation Department of Clinical Practice and Support Hiroshima University Hospital Hiroshima Japan; 3 Bio-environmental Adaptation Sciences Graduate School of Biomedical and Health Sciences Hiroshima University Hiroshima Japan

**Keywords:** gait rehabilitation, Hybrid Assistive Limb, amputation, prosthetics, quality of life, activities of daily living

## Abstract

**Background:**

Lower limb amputation rates are increasing owing to aging and vascular diseases. However, no standardized rehabilitation protocol has been established for regaining walking ability. Conventional rehabilitation delays prosthetic gait training until 6-8 weeks post amputation, prolonging recovery and increasing medical expenses.

**Objective:**

This study aims to evaluate the impact of neurorehabilitation using the Hybrid Assistive Limb (HAL) for Medical Use Lower Limb Type, developed by Japan's Tsukuba University and the robotics company Cyberdyne, on gait acquisition, improvement in activities of daily living (ADL), and enhancement of quality of life (QOL) in patients with lower limb amputation, and assess the effectiveness of early HAL-assisted gait training before and after amputation.

**Methods:**

This single-arm trial will include 20 patients undergoing unilateral transfemoral or transtibial amputations. HAL-assisted gait training will be performed 5 days per week (30 minutes per session) until postoperative week 8. The primary outcome is a 2-minute walking distance, while secondary outcomes include muscle strength, balance, gait parameters, and ADL/QOL measures. Statistical analyses will be conducted using SPSS (IBM Corp), and results obtained at the following time points will be compared: preoperative, postintervention, and 6-month follow-up.

**Results:**

The planned sample size is 20 patients, calculated using JMP version 12 (SAS Institute) based on an expected 1.5-fold improvement in 6-minute walk distance (effect size 0.8, α=.05, power=0.8). Analyses will be performed using SPSS version 15.0 (IBM Corp). The 2-minute walk test (primary outcome) and secondary outcomes will be compared at baseline, post intervention, and 6-month follow-up using 1-way analysis of variance or Kruskal-Wallis test, and *P* values <.05 and 95% CIs will be reported.

**Conclusions:**

This is the first study to apply HAL-assisted neurorehabilitation to patients with lower limb amputations. Early gait training may increase prosthetic gait acquisition rates, shorten rehabilitation and hospitalization periods, and reduce medical costs. If effective, this study may contribute to the development of a Japan-originated rehabilitation program and provide clinical evidence supporting broader HAL implementation for patients with lower limb amputations.

**Trial Registration:**

Japan Registry of Clinical Trials jRCTs062200031; https://jrct.mhlw.go.jp/latest-detail/jRCTs062200031

**International Registered Report Identifier (IRRID):**

PRR1-10.2196/76509

## Introduction

In Japan, 5.4-6.2 individuals per 100,000 population undergo lower limb amputation annually, whereas the rate is higher in Europe and the United States, ranging from 7.4 to 41.3 per 100,000 population [1]. The causes of amputation include trauma from traffic accidents, tumors, congenital diseases, and peripheral circulatory disorders. Notably, circulatory disorder–related amputations are increasing owing to the rising prevalence of conditions, such as diabetes and arteriosclerosis [2]. In a population-based study of geriatrics with vascular amputations, only 36% achieved successful prosthetic fitting, with advanced age and transfemoral amputation linked to failure [3]. Given the anticipated increase in lower limb amputations due to the aging population, medical and social costs, such as long-term hospitalization, rehabilitation, provision of prostheses, and nursing support, are set to escalate [4]. Therefore, restoring the ability to walk and improving the quality of life (QOL) of individuals with lower limb amputations requires immediate attention.

No established method for training patients to walk following lower limb amputation exists [5]. Traditional rehabilitation protocols after lower limb amputation require approximately 8 weeks for residual limb maturation [6], delaying the start of prosthetic gait training until the maturation period is complete. Furthermore, while active prostheses enable a more normalized gait pattern through neuromuscular control [7,8], passive prostheses are typically restricted to use within 8 weeks postamputation, further slowing the acquisition of a new gait pattern using a prosthetic limb. One of the reasons for the lack of an established method for training patients to walk after amputation is that it takes 6-8 weeks for the stump to mature after amputation, and patients cannot bear weight on the amputated side; therefore, they can only walk on the healthy side. Consequently, training is conducted to strengthen the muscles other than the stump, improve function, manage the condition of the stump, and provide functional training on the stump side [9].

The medical robot “HAL (Hybrid Assistive Limb) for Medical Use Lower Limb Type,” developed by Japan's Tsukuba University and the robotics company Cyberdyne, has been reported to improve the walking ability of patients with 10 slowly progressive neuromuscular diseases, such as spinal muscular atrophy and muscular dystrophy [10]. In April 2016, it became the first medical robot in the world to be covered by medical insurance. The HAL detects weak bioelectrical signals on the skin surface when the user tries to move their muscles and assists their movements. In neurorehabilitation using the HAL for Medical Use Lower Limb Type, neuroplasticity is promoted, and walking is improved by repeating accurate walking movements without errors based on the intention of the wearer [11,12]. However, no studies have documented the application of HAL for Medical Use Lower Limb Type in individuals with lower limb amputations.

Therefore, we aimed to investigate the effect of using the medical robot “HAL for Medical Use Lower Limb Type” for walking rehabilitation before and immediately after above-knee and below-knee amputation surgery on independent walking, activities of daily living (ADL), and QOL in individuals with lower limb amputations, and to help establish new clinical applications of the medical robot HAL for individuals with above-knee amputations. It is expected that “the rate of achievement of walking with a prosthetic limb itself may increase” by “being able to start rehabilitation early.” Elucidating the effect of the HAL for Medical Use Lower Limb Type on individuals with lower limb amputations would enable the establishment of a unique early rehabilitation program for lower limb amputation originating in Japan, aimed at the acquisition of walking ability in individuals with lower limb amputations.

## Methods

### Study Setting

The study will be conducted in a university hospital setting. Specifically, it will take place at the Department of Rehabilitation Medicine, Hiroshima University Hospital, Japan. All data will be collected within Japan. A list of trial sites is available upon request from the corresponding author.

For participant recruitment, potential candidates visiting the orthopedic department of the implementing facility will be referred to the rehabilitation department, where a rehabilitation doctor will explain the study. After obtaining written consent from the potential participants, the principal investigator (YM) or coinvestigator (KU and TA) will conduct the necessary screening tests to assess eligibility. The participants will be assessed for eligibility based on the inclusion and exclusion criteria. The principal investigator (YM) or coinvestigator (KU and TA) will record the required information on the case registration form and submit it to the registration center. A research subject identification code will be assigned to each participant. This identification code will be arbitrary and contain no information capable of identifying a specific individual. The principal investigator (YM) or coinvestigator (KU and TA) will retain the registration confirmation slips.

### Eligibility Criteria

The target population will include individuals scheduled for above- or below-knee amputation, and the inclusion and exclusion criteria are described in Textbox 1.

The inclusion criteria are designed to facilitate rehabilitation after amputation during hospitalization at our hospital. Participants are required to understand the purpose of the study and be able to follow instructions on wearing and walking with the HAL. The minimum age is set at 16 years to ensure that participants can independently provide consent. Furthermore, the height and weight requirements are set to accommodate the HAL S and M sizes. Final participant eligibility will be based on the judgment of the doctor.

The exclusion criteria are set to prevent variability due to participants who have experienced traditional amputation rehabilitation and to exclude participants, considering their safety when conducting this study.

**Inclusion and exclusion criteria.**

**Inclusion criteria**

- Patients scheduled to undergo unilateral transfemoral or transtibial amputation at our hospital (underlying disease is not specified)
- Patients capable of providing written informed consent
- Patients aged ≥16 years at the time of consent acquisition
- Patients with a height between 150 and 175 cm
- Patients with a body weight between 40 and 100 kg
- Patients deemed by a physician to have the potential to walk postoperatively with a prosthetic limb
- Patients able to comply with the hospitalization and treatment schedule during the treatment period

**Exclusion criteria**

- Patients with a history of amputation of the contralateral lower limb
- Patients requiring ventilator support, respiratory assistance devices, or oxygen therapy, or those deemed by a physician to require such interventions
- Patients considered unable to participate in walking exercises due to exertional dyspnea, heart failure, or similar conditions
- Patients with severe skeletal deformities (eg, osteoarthritis, spondylosis, and scoliosis) that may hinder walking exercises
- Patients with medical conditions such as bleeding disorders or osteoporosis that could interfere with walking exercises, or those deemed by a physician to be unable to walk postoperatively with a transfemoral or transtibial prosthesis
- Patients with severe hepatic, renal, or cardiovascular diseases
- Patients who are pregnant, may be pregnant, or wish to become pregnant during the study period
- Patients who cannot have HAL-ML05 (developed by Japan's Tsukuba University and the robotics company Cyberdyne) electrodes attached due to dermatological conditions or a short residual limb
- Patients deemed unsuitable for participation in the study by a physician

### Ethical Considerations

This study is conducted in accordance with the Declaration of Helsinki and has received ethical approval from the Ethical Committee for Clinical Research of Hiroshima University (approval no CRB6180006). All participants are provided with detailed information about the study’s aims, procedures, risks, and benefits, and written informed consent is obtained prior to participation. Informed consent will be obtained by trained investigators or clinical staff who are familiar with the study protocol. The consent process will take place in a private setting at the Hiroshima University Hospital, where potential participants (or their legally authorized representatives, if applicable) will receive detailed verbal and written explanations of the study.

Participants will be given sufficient time to ask questions and consider participation. Participants will be clearly informed that participation in ancillary studies is entirely voluntary and will not affect their involvement in the main study. Participants are informed that participation is voluntary and that they may withdraw from the study at any time without consequences. A separate consent form will be provided and signed before any ancillary data or specimen collection.

If participants agree, additional informed consent will be obtained for the collection and use of their data and biological specimens in ancillary studies related to the main trial. These studies may involve future research on rehabilitation outcomes, genetic markers, or biomarkers, and will be subject to approval by the institutional ethics committee.

Personal data are anonymized to maintain confidentiality. Although the study involves physical activities using the HAL device, comprehensive safety protocols are implemented, and medical staff are present during all intervention sessions to promptly manage any adverse events. Previous studies involving HAL report a favorable safety profile, but potential physical risks such as skin irritation, fatigue, and joint discomfort are explained clearly to participants prior to consent.

While no specific clinical trial insurance is arranged, standard institutional liability coverage applies. In the event of study-related adverse events, participants receive immediate medical attention at no personal cost. This coverage is consistent with the policies of the host institution and meets the ethical requirements for investigator-initiated exploratory research. The study is registered in the Japan Registry of Clinical Trials under registration number CRB6180006.

### Interventions

**Figure 1:**
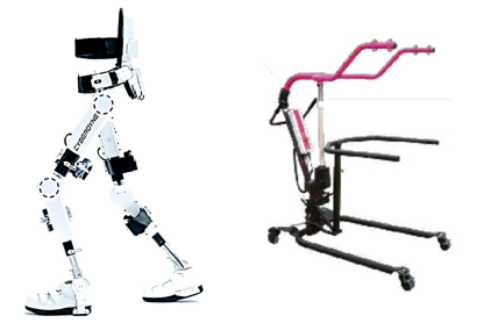
(Left) Hybrid Assistive Limb (developed by Japan's Tsukuba University and the robotics company Cyberdyne) and (right) walker (All-in-One, Ropox A/S).

The intervention device used in this study is the HAL for Medical Use Lower Limb Type (HAL-ML05, Cyberdyne Inc; Medical Device Approval number: 22700BZX00366000; Figure 1). This device was developed for patients with slowly progressive neuromuscular diseases to improve walking function through intermittent use of HAL and repeated performance of gait exercises while assisting lower limb movements based on bioelectric signals.

In addition, for patients with unstable standing posture, a walker (All-in-One, Ropox A/S; Figure 1) will be used as a peripheral device for the HAL to aid walking. This walker features a battery-powered unloading lift, enabling a one-touch adjustment of the unloading weight and accommodating users of various body types and training levels. When used in combination with the HAL Lower Limb Type (NON-MEDICAL), it enables safer gait training.

**Figure 2:**
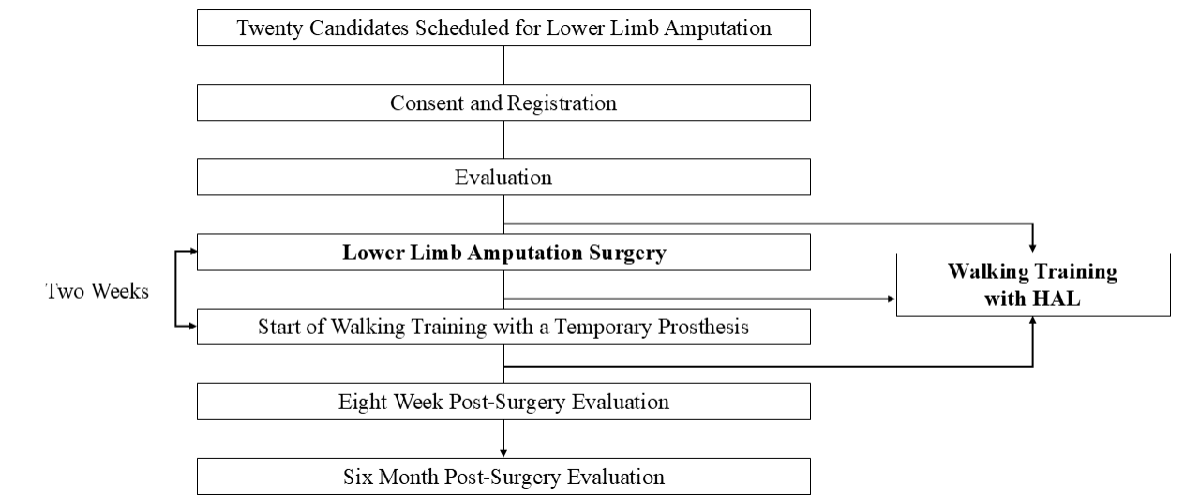
Flowchart for trial. HAL: Hybrid Assistive Limb (developed by Japan's Tsukuba University and the robotics company Cyberdyne).

The intervention outline is shown in Figure 2. An explanation of the study will be provided after an initial examination by the principal or coinvestigator. If the patient agrees to participate, they will be registered as a study participant. Before amputation surgery, preoperative assessments will be conducted, and at least one session of HAL-assisted gait training will be performed. Postoperatively, in addition to general rehabilitation exercises, HAL-assisted gait training will commence after the attending physician approves an increased activity level (mobilization clearance). HAL training will be conducted 5 days a week, with one session daily for 30 minutes. In the early postoperative phase, patients may perform HAL-assisted gait training without a prosthesis. Once the residual limb matures sufficiently and is deemed fit by the physician, a temporary prosthesis—customized for each patient but standardized in terms of structure—will be fitted to begin prosthetic-based gait training. The attending surgeon will determine the initiation of HAL training based on each patient’s postoperative recovery status, particularly the condition of the surgical wound and residual limb. If adverse skin reactions (such as inflammation, abrasions, or irritation at the electrode sites) are observed, HAL training will be suspended temporarily to avoid complications. The HAL device will be used exclusively for overground gait training during each rehabilitation session. Other tasks, such as sit-to-stand practice, orthotic fitting, and outdoor walking training, will be conducted without HAL support.

As the residual limb matures, prosthetic gait training will begin using a temporary prosthesis. Patients who underwent transfemoral amputations will use a transfemoral prosthesis, whereas those who underwent transtibial amputations will use a transtibial prosthesis. The temporary prosthesis is standardized for gait training and will not differ among patients, whereas the socket will be custom-made to fit the residual limb of each patient. HAL-assisted gait training will continue until postoperative week 8. The hospitalization period will last until the completion of the evaluation at postoperative week 8. At this point, all assessment items will be measured, with a final evaluation conducted 6 months postoperatively.

During gait training, patients may experience significant fatigue, inflammation at the electrode site, abrasions at contact points between the HAL and the body, and adverse muscle or joint pain. If any of these occur, training will be immediately discontinued, and the patient will undergo a medical examination.

According to the study protocol, criteria for discontinuing or modifying the allocated intervention for individual participants include the following: withdrawal of informed consent; discovery after enrollment that the participant does not meet the eligibility criteria; worsening of the primary disease that makes use of the study device inappropriate; exacerbation of comorbid conditions that hinders continuation; occurrence of adverse events that make continuation difficult; confirmation of pregnancy; significant noncompliance with study procedures; markedly low frequency of intervention use due to postoperative complications; premature termination of the entire study; or any other reason deemed appropriate by the investigator (YM, KU, and TA).

When discontinuation occurs, the reason will be explained to the participant if necessary, and postdiscontinuation care will be provided to ensure the participant is not disadvantaged.

The principal investigator (YM) considers early termination of the study in cases where the planned number of participants cannot be recruited within the designated period; the ethical review board requires protocol changes that are not acceptable; facts or information are discovered that undermine the ethical or scientific validity of the study; the integrity of the study conduct or results is compromised; or critical information emerges regarding the quality, safety, or effectiveness of the study device.

If the ethics committee recommends or instructs discontinuation, the study will be terminated accordingly. In such cases, the reason for termination will be reported in writing to the hospital director, and a study termination report will be submitted without delay. Notification will also be made to the ethics committee and the Ministry of Health, Labour and Welfare within 10 days of termination.

To enhance participant adherence to the intervention (HAL-assisted gait training), the following strategies will be implemented. At the time of enrollment, participants receive thorough explanations and education regarding the importance and expected benefits of the intervention. All intervention sessions are scheduled at times convenient for the participants to minimize dropout due to logistical reasons. Attendance is monitored through structured session logs, and any missed sessions are followed up with a reminder call or consultation by research staff. A fixed staff member is assigned to each participant to ensure continuity of care and to foster trust and motivation. Additionally, participants receive regular positive feedback based on their performance and engagement to support continued participation.

During the trial period, participants are permitted to receive standard medical care and rehabilitation interventions that do not interfere with the objectives or outcomes of the study. Permitted interventions include routine medications for chronic conditions such as antihypertensives and antidiabetic agents, physical therapy sessions unrelated to the study intervention, and participation in nutritional support or general wellness programs. In contrast, prohibited activities include the introduction of new rehabilitation devices or therapies that may affect outcome measures, participation in other clinical trials during the study period, and any medical or therapeutic interventions deemed by the investigator (YM, KU, and TA) to potentially confound the study results.

All concomitant treatments will be documented and monitored throughout the study to ensure they do not compromise data integrity.

If any physical or psychological harm occurs as a result of participation in this study, no financial compensation will be provided. This policy has been reviewed and approved by the institutional ethics committee and will be clearly explained to all participants during the consent process. Participation in the study will only proceed after obtaining their full understanding and written informed consent.

If any adverse effects or harm are observed, appropriate medical care and treatment will be provided without delay. However, the cost of such care under the national health insurance system will be the participant’s responsibility.

Since gait training using the HAL device for conditions such as stroke and Parkinson disease falls within the scope of routine rehabilitation care and is not considered to exceed the boundaries of standard clinical practice, we have determined that clinical trial insurance is not necessary. Furthermore, no serious adverse events or device malfunctions have been reported in prior clinical research involving HAL.

Participants who choose not to participate in the study will receive standard rehabilitation therapy for patients with lower limb amputation, including exercises targeting the residual limb and other parts of the body.

### Outcomes

All gait evaluations at 2 weeks, 8 weeks, and 6 months postoperatively will be conducted without the use of the HAL device. For the primary outcome, assessment will be conducted at 8 weeks postoperatively to evaluate the short-term effectiveness of early HAL-assisted gait training, which is expected to influence the critical period of initial rehabilitation and prosthetic adaptation. Functional recovery may continue beyond this period, particularly up to 6 months; however, the 8-week period was chosen as the primary end point to capture the immediate impact of the intervention. A 6-month follow-up will provide important secondary data on longer-term outcomes. At each time point, walking ability will be assessed under standard conditions using the residual limb or temporary prosthesis, as applicable. A comprehensive examination of various walking-related factors is necessary; therefore, we will collect the items listed in Table 1 extensively. Among these evaluation items, the 2-minute walking distance [13,14] is set as the primary evaluation item, as it is a general indicator of walking ability and is directly linked to ADL and QOL. Other items will be collected as secondary evaluation items. The Locomo 25 is an assessment tool used to evaluate the condition of locomotive syndrome, which indicates a decline in mobility [15].

**Table 1:** Primary and secondary outcomes.

Assessment item			Description
**Physical function**			
	2-minute walking distance	Measures the total distance walked in 2 minutes, with pylons placed at 30-m intervals.	
	Bilateral lower limb muscle strength	Measured using μ-Tas (ANIMA Corporation) under isometric contraction conditions.	
	Skeletal muscle mass	Measured using InBody S10.	
	Sensory	Evaluated using a tactile sensory testing device.	
	Range of motion	Assessed using the Tokyo University–style goniometer.	
	Berg Balance Scale	Evaluates balance ability using a validated assessment form.	
	Short Physical Performance Battery	A useful measure combining balance ability and walking performance, assessed using a standard evaluation form.	
	Gait analysis	Measures time to complete the TUG and 10-m walk. Gait pattern is analyzed during the 10-m walk using the PhysiGait (CREACT Co, Ltd) gait analysis system, evaluating step length, stride length, weight distribution, gait speed, etc.	
	Cardiopulmonary function	Maximal oxygen consumption (VO₂ max): Measured using a respiratory gas analyzer.	
	Locomo 25	A questionnaire consisting of 25 items regarding body pain and difficulties in daily activities over the past month. Responses are scored on a 5-point scale (0-4 points per item). Locomotive Syndrome Grade 1: Total score ≥7 points. Locomotive Syndrome Grade 2: Total score ≥16 points.	
	Muscle activity	Electromyography (EMG): Measures muscle activity distribution patterns using a multichannel surface EMG system.	
Activity			BI (Barthel Index), FIM (Functional Independence Measure), FAI (Frenchay Activities Index).
Participation			SF-36, Health Utilities Index, Euro-Qol-5D-5L, Long-Term Care Insurance Certification Level, General Self-Efficacy Scale, WHODAS2.0, Locomotor Capabilities Index (LCI), Prosthetic Evaluation Questionnaire Japan.

All physical function assessments (2-minute walk test) will be conducted without HAL assistance.

In addition, patient background information, including sex, date of birth, age, hospitalization status (inpatient or outpatient), height, weight, comorbidities, past medical history, current medical history, medication status, and residual limb length, will be recorded. Vital signs, such as body temperature, blood pressure, pulse rate, and respiratory rate, will also be documented. Furthermore, blood biochemical tests, including white blood cells, red blood cells, hemoglobin, platelets, creatine kinase, albumin, and C-reactive protein, as well as physiological test results, such as electrocardiogram and spirometry, will be collected. The presence or absence of adverse events, including contact dermatitis, abrasions, muscle damage due to overload, and other adverse events, will also be recorded.

The time schedule for participant enrollment, interventions (including pre- and postoperative periods), assessments, and follow-up visits is summarized in Table 2. This table is structured in accordance with the SPIRIT (Standard Protocol Items: Recommendations for Interventional Trials) 2013 statement and outlines key time points, such as preobservation, surgery, and follow-up phases (2 weeks, 8 weeks, and 6 months postoperatively).

The SPIRIT schedule is presented in table format in the manuscript and is provided to all participants as part of the informed consent process. The schedule includes key components such as enrollment procedures, including informed consent and baseline data collection; interventions, including HAL gait training and prosthesis-based rehabilitation; outcome assessments, which consist of physical function measures, blood biochemistry, and physiological tests; and safety monitoring, including adverse event tracking throughout all phases of the study.

All assessments and interventions are scheduled to ensure consistency and patient safety throughout the clinical trial.

**Table 2:** Schedule of enrollment, interventions, assessments, and visits (SPIRIT^a^-compliant).

Study period	Enrollment	Preobservation	Day 0	2 weeks postoperative	8 weeks postoperative	6 months postoperative	Withdrawal
Informed consent	✓						
Participant characteristics	✓						
Physical function^b^		✓			✓	✓	
Activities^c^		✓			✓	✓	
Participation^d^		✓			✓	✓	
Blood biochemistry^e^		✓			✓	✓	
Physiological tests^f^		✓			✓	✓	
HAL^g^ gait training^h,i^		✓^h^		✓^i^	✓^i^		
Prosthesis gait training^j^					✓^j^	✓	
Adverse event monitoring		✓	✓	✓	✓	✓	✓

^a^SPIRIT: Standard Protocol Items: Recommendations for Interventional Trials.

^b^All physical function assessments (2-minute walk test) will be conducted without HAL assistance.

^c^Activity assessments such as gait tests and activities of daily living (ADL) scales.

^d^Participation measures (social participation scales).

^e^Blood tests: standard biochemical profile.

^f^Physiological evaluations (electrocardiogram and respiratory tests).

^g^HAL: Hybrid Assistive Limb (developed by Japan's Tsukuba University and the robotics company Cyberdyne).

^h^Initial HAL training session presurgery.

^i^Regular HAL training post surgery.

^j^Rehabilitation using a temporary prosthesis.

### Sample Size

The calculation was performed with α=.05, a statistical power of 0.8 (80%), and an effect size of 0.8, resulting in a total sample size of 20. The target sample size includes a margin to accommodate potential dropouts. However, if the number of dropouts significantly impacts statistical power or data integrity, we plan to recruit additional participants to maintain the robustness of the analysis.

The sample size will be estimated using data from the 6-minute walk distance (6MWD), as sufficient effect size data for this outcome are available in the literature, and it is a widely recognized measure of functional walking capacity in rehabilitation research. However, performing the full 6MWD may be overly demanding for patients in the early postoperative period following limb amputation; therefore, the 2-minute walk distance (2MWD) will be selected as the primary end point. The 2MWD has been validated as a reliable and less physically demanding alternative to the 6MWD, with strong correlations reported between the two measures in neuromuscular and rehabilitation populations [13,15].

### Statistical Analysis

The target sample size was set at 20 patients scheduled for lower limb amputation. Using JMP version 12 (SAS Institute), the sample size was calculated based on a research design assuming that the 6MWD would improve by a factor of 1.5, referencing previous studies on gait analysis using the HAL [16]. The calculation was performed with α=.05, power=0.80, and a prespecified effect size.

The primary analysis population for efficacy evaluation will be the Full Analysis Set (FAS), whereas the secondary analysis population will be the Per Protocol Set (PPS).

The FAS includes all study participants who have undergone at least one session of HAL-assisted gait training and have had at least one efficacy-related observation thereafter. However, individuals who fail to meet the eligibility criteria will be excluded. The PPS comprises participants from the FAS who have completed HAL-assisted gait training (5 days per week, 30 minutes per day) without major deviations from the study protocol and for whom the primary evaluation item can be assessed.

Statistical analyses will be conducted using SPSS version 30.0 (IBM Corp). The primary evaluation item, the 2-minute walking distance, will be analyzed using the FAS population, and the mean (SD) [or median (minimum-maximum)] will be calculated. Comparisons between preoperative, postintervention, and 6-month postoperative outcomes will be performed using a 1-way ANOVA or the Kruskal-Wallis test, with a 2-sided significance level of 5%, and a 95% CI will be calculated. In addition, the same analysis will be performed using the PPS as a secondary approach.

For secondary evaluation items, analysis will also be conducted using the FAS population. The mean (SD) [or median (minimum-maximum)] will be calculated for bilateral lower limb muscle strength, skeletal muscle mass, sensory impairment, range of motion, Berg Balance Scale, timed up and go test, gait parameters (step length, stride length, weight distribution, and gait speed during the 10-minute walk test), maximal oxygen uptake, and Locomo 25. Comparisons between preoperative, postintervention, and 6-month postoperative outcomes will be performed using a 1-way ANOVA or the Kruskal-Wallis test, and multiple comparisons will be conducted using the Tukey method (or Steel-Dwass test). A 2-sided significance level of 5% and a 95% CI will be used. The same analysis will also be performed using the PPS as a secondary approach.

Furthermore, for Barthel Index (BI), Functional Independence Measure (FIM), Frenchay Activities Index (FAI), SF-36, Health Utilities Index, EQ-5D-5L (quality-adjusted life years), long-term care insurance certification level, General Self-Efficacy Scale, and WHODAS 2.0, the mean (SD) [or median (IQR)] will be calculated. Comparisons between preoperative, postintervention, and 6-month postoperative outcomes will be performed using a 1-way ANOVA or the Kruskal-Wallis test, with multiple comparisons conducted using the Tukey method (or Steel-Dwass test). A 2-sided significance level of 5% and a 95% CI will be calculated. The same analysis will be conducted using the PPS as a secondary approach.

The primary efficacy analysis will be conducted using the FAS, which includes all participants who received at least one session of HAL-assisted gait training and have at least one postintervention efficacy observation. Participants who are found to violate key eligibility criteria will be excluded from the FAS.

A secondary analysis will be performed using the PPS, which is a subset of the FAS consisting of participants who completed HAL training (5 days per week, 30 minutes per session) without any major protocol deviations and for whom the primary outcome data are available.

For missing data, a descriptive analysis will first be conducted to assess the pattern and mechanism of missingness. If the data are judged to be missing at random, multiple imputation using chained equations will be applied to handle missing values in the primary outcomes. Additionally, sensitivity analyses will be performed by comparing results between complete-case and imputed datasets to evaluate the robustness of the findings.

## Results

### Sample Size and Participant Flow

Based on previous studies of gait analysis using HAL, we calculated the required sample size using JMP (version 12; SAS Institute), assuming a 1.5-fold improvement in the 6MWD. With an alpha of .05, a power of 0.8, and an effect size of 0.8, the calculated sample size was 20. Considering potential dropouts, we set the final target sample size at 20 participants. The overall flow of the trial, including participant recruitment, consent, evaluation, surgery, intervention, and 6-month follow-up, is planned in accordance with the SPIRIT 2013 statement.

### Planned Outcomes and Statistical Analysis

For the primary outcome, the 2MWD, we will compare results at 3 time points: preoperative, post intervention, and 6-month follow-up. Data will be presented as mean (SD) [or median (minimum-maximum)]. Comparisons will be performed using a 1-way ANOVA or the Kruskal-Wallis test, with a *P* value <.05 and 95% CIs reported.

Secondary outcomes will be analyzed using a similar approach. Descriptive statistics will be calculated for objective measures, like muscle strength, balance, and gait parameters, as well as for subjective scales including ADL/QOL assessments (BI, FIM, and FAI). Multiple comparisons will be conducted using the Tukey method or the Steel-Dwass test, with a *P* value <.05 and 95% CIs also reported.

For missing data, we will first conduct a descriptive analysis to assess the pattern of missingness. If the data are judged to be missing at random, multiple imputation will be applied to the primary outcomes. Sensitivity analyses will also be performed by comparing results from complete-case and imputed datasets to assess the robustness of the findings.

The primary efficacy analysis will be conducted on the FAS, and a secondary analysis on the PPS.

## Discussion

### Principal Findings

This study represents the first attempt at using the HAL for Medical Use Lower Limb Type for neurorehabilitation in individuals with lower limb amputations and also evaluates its effectiveness in early postoperative gait training. By enabling the early initiation of rehabilitation, the success rate of prosthetic gait acquisition is expected to increase. In conventional rehabilitation for individuals with lower limb amputations, active gait training is difficult during the 6-8 weeks required for residual limb maturation, leading to delays in the start of prosthetic walking. We aimed to address this issue by introducing HAL-assisted gait training from an early postoperative stage, with the expectation that it will improve the success rate of prosthetic gait acquisition, shorten the postoperative rehabilitation and hospitalization periods, enhance ADL and QOL, and contribute to medical cost reduction and efficiency. From academic and scientific perspectives, no prior reports on robotic movement therapy and neurorehabilitation based on voluntary movements in individuals with lower limb amputations exist. Consequently, this study will present the first reported findings, highlighting its academic originality and significance.

### Comparison With Prior Work

Early neurorehabilitation may facilitate the smooth acquisition of walking patterns. Previous studies on HAL-assisted gait training in patients with cerebrovascular disease have shown that repetitive, precise walking movements help maintain and improve muscle strength and balance ability [16,17]. In addition, early initiation of prosthetic gait training has been reported to enable independent walking even in older women [18]. Thus, implementing early gait training may promote recovery of physical function within the first 8 weeks of postoperative rehabilitation. Furthermore, studies have shown that patients who used a training prosthesis during rehabilitation had shorter hospital stays compared with those who did not [19,20].

### Implications for Clinical Practice and Policy

If early HAL-assisted gait training successfully enhances physical function and enables independent walking, it may lead to earlier hospital discharge and reduced medical costs. Currently, no standardized rehabilitation protocol from lower limb amputation to prosthetic gait acquisition exists [5]. If the results of this study prove effective, they may serve as a foundation for establishing a novel rehabilitation program in Japan. Moreover, HAL is currently covered by medical insurance for specific neuromuscular diseases [10], and the potential effectiveness of this study could provide scientific evidence to support the expansion of this insurance to cover individuals with lower limb amputations, potentially benefiting a broader patient population.

### Safety Considerations

Previous clinical studies have demonstrated that HAL has a favorable safety profile, with no serious adverse events reported and only minor and transient effects such as skin irritation, pressure discomfort, or fatigue [10,21]. In this study, if complications occur, specific management strategies will be applied. Skin irritation will be managed with local skin care and adjustment of electrode placement or harness fitting. Muscle fatigue will be addressed by reducing training intensity and duration or by introducing longer rest intervals. Joint pain or discomfort will lead to the temporary suspension of training followed by a medical evaluation. HAL training will only be resumed once the attending physician confirms that continuation is safe.

### Limitations

A key limitation of this study is the absence of a control group, which restricts the ability to attribute observed improvements directly to HAL-assisted rehabilitation. Moreover, the absence of a conventional rehabilitation control group limits the ability to attribute improvements solely to the HAL intervention. The single-arm design is appropriate for an exploratory investigation; however, future randomized controlled trials will be necessary to confirm efficacy and compare HAL-assisted rehabilitation with conventional rehabilitation protocols.

Second, the sample size is limited to 20 participants. This decision reflects the exploratory nature of the study and anticipates substantial individual variability in residual limb characteristics and rehabilitation backgrounds among transfemoral amputees. Third, the application of HAL is subject to technical constraints related to patient physique and residual limb condition, meaning it cannot currently be applied to all individuals with lower limb amputations. These limitations, arising from narrow eligibility criteria (eg, height, weight, and residual limb conditions), may restrict the generalizability of the findings. However, such strict criteria are necessary to ensure the safe and effective use of the HAL system, which requires specific physical and anatomical conditions for proper fitting and functionality. As the technology evolves, future studies may consider broader inclusion criteria to enhance applicability to the wider amputee population. Ultimately, this research will contribute to the development of guidelines for early rehabilitation following lower limb amputation and facilitate multicenter collaborative studies to strengthen the evidence base.

The 6-month follow-up assessment may be less sensitive in detecting differences attributable to early intervention timing; however, it is intended as a final confirmation of functional walking status. The primary outcome, however, is the 2MWD measured immediately after the 8-week intervention period, which directly reflects the effect of early HAL-assisted rehabilitation. We acknowledge that a 6-month follow-up period may not capture the full extent of functional recovery, particularly for transfemoral amputees. In particular, longer-term adaptations such as neuromuscular reorganization, prosthetic control proficiency, and reintegration into daily activities may require extended periods to fully manifest. While this duration allows for the assessment of short- to midterm effects of early intervention, the decision to limit follow-up to 6 months balances practical feasibility, participant burden, and the exploratory nature of this protocol. Future studies should consider longer follow-up periods to evaluate sustained outcomes and late-phase rehabilitation progress.

Another limitation is the strict eligibility criteria required for HAL use, including specific height, weight, and residual limb conditions. These requirements may exclude certain patient populations, which could limit the generalizability of the findings. Nevertheless, these criteria are necessary to ensure patient safety and to accommodate the technical specifications of the HAL system.

### Conclusions

Early postoperative HAL-assisted gait training can increase the success rate of prosthetic walking, shorten the rehabilitation period, and reduce medical costs. The findings of this study are expected to contribute to the establishment of a standardized rehabilitation program originating from Japan for individuals with lower limb amputations and also serve as a foundation for novel clinical applications of HAL.

## Abbreviations

2MWD: 2-minute walk distance
6MWD: 6-minute walk distance
ADL: activities of daily living
BI: Barthel Index
FAI: Frenchay Activities Index
FAS: Full Analysis Set
FIM: Functional Independence Measure
HAL: Hybrid Assistive Limb
PPS: Per Protocol Set
QOL: quality of life
SPIRIT: Standard Protocol Items: Recommendations for Interventional Trials

## Data Availability

The datasets generated or analyzed during this study are not publicly available due to privacy restrictions, but are available from the corresponding author upon reasonable request. Requests for access to the data should include the purpose of the request and will be subject to approval by the ethics committee of Hiroshima University Hospital.

## References

[1] Narres M, Kvitkina T, Claessen H, Droste S, Schuster B, Morbach S, Rümenapf G, Van Acker K, Icks A (2017). Incidence of lower extremity amputations in the diabetic compared with the non-diabetic population: a systematic review. PLoS One.

[2] Barbosa BMB, Monteiro R, Sparano L, Bareiro R, Passos A, Engel E (2016). Incidence and causes of lower-limb amputations in the city of Ribeirão Preto from 1985 to 2008: evaluation of the medical records from 3,274 cases. Rev Bras Epidemiol.

[3] Fletcher D D, Andrews K, Butters M, Jacobsen S, Rowland C, Hallett J (2001). Rehabilitation of the geriatric vascular amputee patient: a population-based study. Arch Phys Med Rehabil.

[4] Jordan RW, Marks A, Higman D (2012). The cost of major lower limb amputation: a 12-year experience. Prosthet Orthot Int.

[5] Highsmith M J, Andrews C, Millman C, Fuller A, Kahle J, Klenow T, Lewis KL, Bradley RC, Orriola JJ (2016). Gait training interventions for lower extremity amputees: a systematic literature review. Technol Innov.

[6] Koonalinthip N, Sukthongsa A, Janchai S (2020). Comparison of removable rigid dressing and elastic bandage for residual limb maturation in transtibial amputees: a randomized controlled trial. Arch Phys Med Rehabil.

[7] Herr HM, Grabowski AM (2012). Bionic ankle-foot prosthesis normalizes walking gait for persons with leg amputation. Proc Biol Sci.

[8] Thatte N, Geyer H (2016). Toward balance recovery with leg prostheses using neuromuscular model control. IEEE Trans Biomed Eng.

[9] Nolan L (2012). A training programme to improve hip strength in persons with lower limb amputation. J Rehabil Med.

[10] Nakajima T, Sankai Y, Takata S, Kobayashi Y, Ando Y, Nakagawa M, Saito T, Saito K, Ishida C, Tamaoka A, Saotome T, Ikai T, Endo H, Ishii K, Morita M, Maeno T, Komai K, Ikeda T, Ishikawa Y, Maeshima S, Aoki M, Ito M, Mima T, Miura T, Matsuda J, Kawaguchi Y, Hayashi T, Shingu Ma, Kawamoto H (2021). Cybernic treatment with wearable cyborg Hybrid Assistive Limb (HAL) improves ambulatory function in patients with slowly progressive rare neuromuscular diseases: a multicentre, randomised, controlled crossover trial for efficacy and safety (NCY-3001). Orphanet J Rare Dis.

[11] Sankai Y, Kaneko M, Nakamura Y (2010). HAL: Hybrid Assistive Limb based on cybernics. Robotics Research. Springer Tracts in Advanced Robotics, vol 66.

[12] Sankai Y, Suzuki K, Hasegawa Y (2014). Cybernics: Fusion of Human, Machine and Information Systems.

[13] Zanudin A, Khong YY, Chong LF, Mohamad NA (2021). Test-retest reliability and construct validity of two-minute walk test in children and adolescents with cerebral palsy. Walailak J Sci Tech.

[14] Kobayashi K, Ando K, Tsushima M, Machino M, Ota K, Morozumi M, Tanaka S, Kanbara S, Ishiguro N, Hasegawa Y, Imagama S (2019). Predictors of locomotive syndrome in community-living people: a prospective five-year longitudinal study. Mod Rheumatol.

[15] Brooks D, Parsons J, Hunter J, Devlin M, Walker J (2001). The 2-minute walk test as a measure of functional improvement in persons with lower limb amputation. Arch Phys Med Rehabil.

[16] Pin TW, Choi H (2018). Reliability, validity, and norms of the 2-min walk test in children with and without neuromuscular disorders aged 6-12. Disabil Rehabil.

[17] Watanabe H, Goto R, Tanaka N, Matsumura A, Yanagi H (2017). Effects of gait training using the Hybrid Assistive Limb® in recovery-phase stroke patients: a 2-month follow-up, randomized, controlled study. NeuroRehabilitation.

[18] Wall A, Borg J, Palmcrantz S (2015). Clinical application of the Hybrid Assistive Limb (HAL) for gait training: a systematic review. Front Syst Neurosci.

[19] Iwasa S, Uchiyama Y, Kodama N, Koyama T, Domen K (2021). Regaining gait using an early postoperative hip prosthesis: a case report of an elderly woman. Prog Rehabil Med.

[20] Pelzer D, Beaudart C, Bornheim S, Maertens de Noordhout B, Schwartz C, Kaux JF (2024). Outcomes of patients with lower limb loss after using a training prosthesis: a retrospective case series study. Healthcare (Basel).

[21] Takahashi K, Mutsuzaki H, Mataki Y, Yoshikawa K, Matsuda M, Enomoto K, Sano K, Kubota Aoi, Mizukami M, Iwasaki N, Yamazaki M (2018). Safety and immediate effect of gait training using a Hybrid Assistive Limb in patients with cerebral palsy. J Phys Ther Sci.

